# Multiscale Radiobiological Assessment of Laser-Driven Very High Energy Electrons Versus Conventional Electrons

**DOI:** 10.1016/j.adro.2026.102028

**Published:** 2026-03-27

**Authors:** Camilla Giaccaglia, Emilie Bayart, Maxime Dubail, Chaitanya Varma, Sophie Heinrich, Julien Gautier, Amar Tafzi, Olena Kononenko, Jean-Philippe Goddet, Jeevan Babu Amakkattu, Isabelle Lamarre-Jouenne, Charles Fouillade, Alessandro Flacco

**Affiliations:** a Laboratoire d'Optique Appliquée (LOA), CNRS, Ecole polytechnique, ENSTA, Institut Polytechnique de Paris, Palaiseau, France; b Institut Curie, Inserm U1021-CNRS UMR 3347, Paris Saclay University, Centre Universitaire, Orsay Cedex, France; c Laboratoire d’Optique et Biosciences, CNRS, INSERM, École polytechnique, Institut Polytechnique de Paris, Palaiseau, France

## Abstract

**Purpose:**

This study systematically investigates the radiobiological effects of very high energy electrons (VHEE) generated by a laser-plasma accelerator (LPA), in comparison with conventional intermediate energy electrons (CIEE) from a conventional linear accelerator. Using in vitro, ex vivo, and in vivo models, we evaluate and compare their potential toxicity on healthy tissues.

**Methods and Materials:**

Cell viability, tissue response, and developmental toxicity were assessed across 3 biological models. In vitro, hTERT-immortalized, human fibroblasts (MRC5-hTERT) were used to generate postirradiation viability-based survival curves following a dose escalation of 2 to 10 Gy. Ex vivo, mouse precision-cut lung slices were analyzed for radiation-induced inhibition of cell replication after 3, 6, and 9 Gy. In vivo, zebrafish embryos were irradiated with 6 and 9 Gy and subsequently evaluated for developmental toxicity through body length and spinal curvature measurements. VHEE irradiations were delivered using the Salle Jaune LPA (Laboratoire d'Optique Appliquée), generating a broad-spectrum electron beam with a mean energy of 140.6 MeV at the biological target, while CIEE irradiations were performed using a conventional 7 MeV linear accelerator (Institut Curie).

**Results:**

In vitro, MRC5-hTERT cells showed no significant difference in radiosensitivity between VHEE and CIEE conditions, with comparable $D_{10}$ values (*P* value =.7). In the ex vivo model, both beams induced a dose-dependent decrease in cell division with no significant interbeam differences at any dose level (*P* value >.99). In vivo, zebrafish embryos exhibited dose-dependent body shortening and increased spinal curvature following both VHEE and CIEE exposure. No significant differences were observed between the 2 modalities at matched doses for any measured metric (*P* value ≥.5).

**Conclusions:**

To our knowledge, this study presents the first multiscale radiobiological evaluation of a laser-driven VHEE beam across cellular, tissue, and organismal levels. Under the investigated conditions, VHEE and CIEE irradiations produce similar biological toxicity. These findings support the feasibility and potential of VHEE generated by LPA for future clinical applications.

## Introduction

The use of very high energy electrons (VHEE) for radiation therapy was first proposed by Des Rosiers et al,^1^ who demonstrated through Monte Carlo simulations their potential to deliver doses to deep-seated tumors with improved conformality. Unlike conventional electron beams (5−25MeV), which are limited to superficial treatments,^2^ VHEE beams (50−300MeV) can penetrate deeply, enabling treatment of a wider range of malignancies. They also exhibit a sharper lateral penumbra than conventional electrons, and even photons at shallow depths, and may enable uniform dose distribution at depth when delivered in opposed-beam configurations.^1^^,^^3^ These early simulations suggested that VHEE beams are less sensitive to tissue inhomogeneities compared with ion beams and photons, a characteristic later confirmed experimentally by Lagzda et al.^4^ This makes them particularly attractive for the treatment of tumors in heterogeneous regions such as the lungs, intestine, or cervix. Recent treatment planning studies further support their clinical potential, with VHEE treatment plans achieving dose distributions comparable or superior to volumetric modulated arc therapy in pediatric, lung, and prostate cancer cases.^5^ From a technological perspective, VHEE beams can be electromagnetically focused and steered with quadrupole magnets, allowing dynamic control of beam trajectory and dose distribution.^6^^,^^7^ This capability would allow for rapid, flexible, multidirectional dose delivery, potentially offering operational advantages compared to conventional photon-based systems.^8^ Despite their promising clinical potential, radiobiological characterization of VHEE beams remains limited. Most experimental studies to date have focused on in vitro assessments of relative biological effectiveness,^9^, ^10^, ^11^ conducted using radiofrequency (RF) linear accelerators (LINACs), which remain the standard technology for generating VHEE.^12^, ^13^, ^14^ However, these RF-based systems require a large footprint and substantial shielding, and are rarely integrated with biological laboratories, thus complicating routine radiobiological studies.

To overcome these limitations, laser-plasma accelerators (LPAs) have emerged as a promising alternative for VHEE generation.^15^ By sustaining gigavolt-per-meter accelerating gradients over millimeter-scale distances, LPAs enable the development of more compact systems that simplify radioprotection requirements and facilitate potential integration into hospital-scale environments. Their compactness further enables novel delivery concepts, including mobile or robotic platforms. For example, Nakajima et al^16^ proposed a robotic, compact gantry-mounted LPA system capable of delivering 50to250MeV electron beams for image guided, intensity modulated radiation therapy. Several studies have also explored the feasibility of LPA-based radiation therapy from a dosimetric perspective, including Monte Carlo simulations of dose deposition.^17^, ^18^, ^19^ Nonetheless, although LPAs offer promising solutions to the spatial and technical constraints of RF systems, most current facilities are still tailored for fundamental physics and lack the biological infrastructure needed for routine radiobiological studies. As a result, only a few radiobiological investigations involving laser-driven VHEE beams have been reported to date.^20^, ^21^, ^22^

In this study, to our knowledge, we report the first multimodel investigation of laser-driven VHEE effects across in vitro, ex vivo, and in vivo assays, using an LPA system adapted for radiobiological research. Endpoints were selected to probe healthy tissue response at different levels: cell viability in human fibroblasts, replication in organotypic mouse lung slices, and developmental toxicity in zebrafish embryos. To ensure clinical relevance and allow benchmarking, all experiments were repeated using a 7 MeV conventional intermediate energy electron (CIEE) accelerator. This study thus establishes a novel preclinical framework for evaluating the radiobiological impact of emerging laser-driven VHEE sources in comparison with clinically established electron modalities.

## Methods and Materials

### Ethics statement

All experimental protocols involving animals complied with French and European regulations on animal care and use (French Decree 2013-118 and EC Directive 2010/63/EU). Zebrafish were obtained from the Laboratory for Optics and Biosciences and handled in accordance with ethical guidelines. Female C57BL/6J mice (6-10 weeks old) were obtained from Charles River Laboratories and housed at the Institut Curie's animal facility.

### Beam generation and irradiation setup

**Figure 1:**
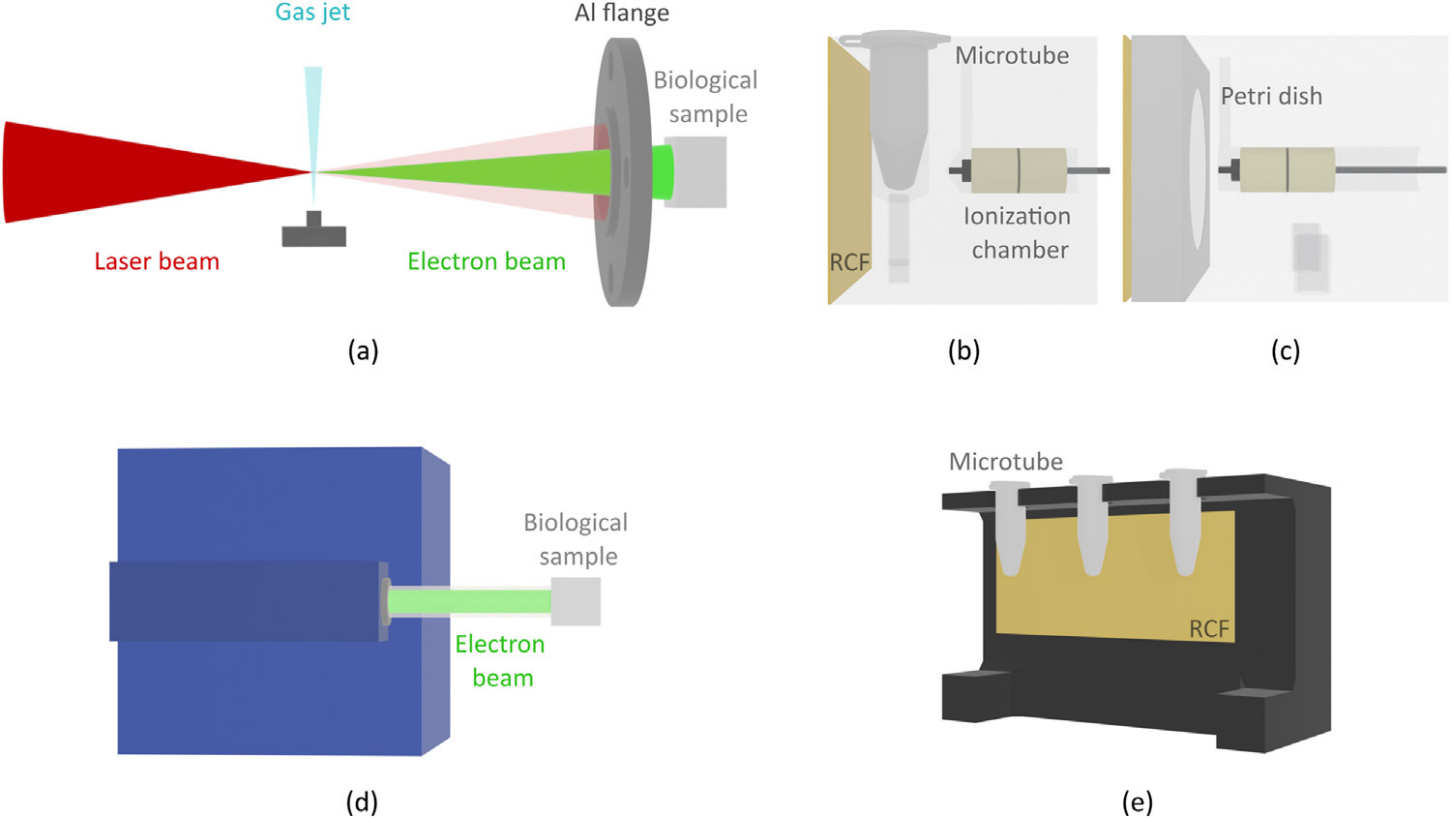
Experimental setups and sample holders used for biological irradiations. Top row (a-c): laser-plasma-accelerated VHEE setup. (a) Scheme of the LPA system: the laser is focused onto a He/N_2_ (2%) gas mixture jet, generating the VHEE beam (green). Electrons exit the vacuum chamber through a 1.5-mm-thick Al flange toward the biological target. (b) In vitro/in vivo holder with microtube positioned between an RCF and an ionization chamber for online dose monitoring. (c) Ex vivo holder with Petri dish support, RCF, and ionization chamber. Bottom row (d-e): LINAC-based CIEE setup. (d) Representation of the conventional 7 MeV LINAC. (e) In vitro/in vivo holder with microtubes placed in front of an RCF, used as dose reference. *Abbreviations*: Al = aluminum; CIEE = conventional intermediate energy electrons; LINAC = linear accelerator; LPA = laser-plasma accelerator; RCF = radiochromic films; VHEE = very high energy electrons.

The VHEE beam was generated using the Salle Jaune laser system, a 60 TW Ti:Sapphire laser operating at the Laboratoire d'Optique Appliquée. The system delivers 30 fs (full width at half maximum) laser pulses with energies exceeding 1.5 J at the focal spot, at a repetition rate of 0.5 Hz. Electrons are accelerated inside a vacuum experimental chamber by focusing the laser onto a gas jet of helium (He) mixed with 2% nitrogen ($N_{2}$), flowing from a 4mm×250μm rectangular nozzle (Fig. 1a). The acceleration conditions were optimized to maximize the beam charge above 50 MeV and to ensure stability in both dose and pointing. For biological irradiation, the beam exits the vacuum chamber through a 1.5-mm aluminum (Al) flange, which maintains the air-vacuum separation, and propagates through air to the target. Under these conditions, the generated broad-spectrum beam is predominantly concentrated in the 50to300MeV range, with an overall angular divergence of (69.3±2.0) mrad at the target. According to Monte Carlo (Geant4) modeling of the irradiation setup, the effective spectral hardening on transition to air yields a dose-weighted average energy of 140.6 MeV within the region of interest at the target plane. The full spectral distribution and dose-weighting curve are provided in Figure E1. Dedicated sample holders (Fig. 1b, c) were used to ensure reproducible positioning and alignment of the biological samples. The number of laser pulses, and consequently the number of dose fractions, was adjusted according to the prescribed dose, while the mean dose rate was maintained at ≈12Gy/min at 0.5 Hz across all experiments.

The CIEE beam was generated using the ElectronFLASH LINAC (SIT S.p.A., RD Dept.) at Institut Curie. The electron energy was set to 7 MeV, and samples were irradiated at a source-to-surface distance of 1.1 m through a 120 mm inner-diameter poly-methylmethacrylate applicator. A 3-mm-thick poly-methylmethacrylate box, filled with water, was attached to the applicator, and the sample holder was fixed to the wall. The holder securely positioned the microtubes containing the biological samples at a distance of 5 mm from the wall, ensuring consistent and uniform dose delivery. A fixed pulse repetition rate of 10 Hz was used, delivering a mean dose rate of approximately ≈30Gy/min for all experiments. The LINAC system is shown in Fig. 1d, while Fig. 1e illustrates the custom holder used for in vitro and in vivo irradiation. Ex vivo samples were irradiated using standard culture plates.

### Dosimetry

During VHEE irradiations, an ionization chamber (Razor Nano Chamber, IBA Dosimetry) was used for real-time dose monitoring. Before each experiment, a calibration was performed to determine the ratio between the dose at the sample position and at the chamber's placement during irradiation. This allowed estimation of the dose-per-pulse and calculation of the required number of shots to reach the prescribed dose. In addition, 1-cm-thick water-equivalent material bricks were placed in front of the sample to adjust the beam size and average dose rate. Real-time readings from the chamber were used to monitor dose delivery and ensure accurate irradiation of the target.

For CIEE irradiations, the LINAC's internal monitoring ionization chamber was calibrated to relate Monitor Units to absolute dose. The LINAC was programmed to automatically stop beam delivery once the desired dose was reached, based on the cumulative Monitor Units.

In both setups, EBT3 radiochromic films (RCFs) (Gafchromic, Ashland) were placed in front of and/or behind the samples to verify the actual dose delivered during each experiment. Films were absolutely calibrated using the 7 MeV electron beam of Institut Curie following the procedure reported in by Giuliano et al.^23^ Dose distributions recorded by RCFs were used to calculate dose uncertainties, which were then propagated across experimental replicates and reported consistently across all figures and analyses. For VHEE irradiations, the dominant contribution to dose uncertainty arose from the transverse beam profile, reflecting variations across the beam size and quantified using RCF measurements, while longitudinal (depth-dependent) variations were negligible. In contrast, for CIEE irradiations, the main source of error was longitudinal dose variation, estimated by Monte Carlo simulations and experimentally validated by longitudinal RCF measurement under identical irradiation conditions, yielding an overall uncertainty of 4%.

### In vitro cell viability assay

The immortalized MRC5-hTERT human fibroblast cell line was cultured in Dulbecco's Modified Eagle Medium (DMEM + GlutaMAX, Gibco), supplemented with 10% fetal calf serum (PAA Laboratories) and Pen-Strep antibiotics (10U/mL penicillin, 10μg/mL streptomycin, Gibco), as described in the established protocol by Bayart et al.^24^ Cells were maintained as monolayers at $37^{\circ }C$ in a humidified atmosphere with $5\%\;CO_{2}$. For irradiation, $∼10^{4}$ cells were transferred into Eppendorf tubes (2 mL for VHEE, 0.5 mL for CIEE) containing growth medium, centrifuged, and irradiated at room temperature ($22-24{\;}^{\circ }C$) with doses ranging from 2 to 10 Gy. Irradiations were performed across 3 independent experimental replicates on separate days. For each replicate, the irradiated cell pellet for each dose condition was resuspended and evenly seeded into 4 wells of a 12-well plate. Fresh medium was added to each well to reach a final volume of 2 mL. After incubation for 5 generations (6 days), cells were harvested using 0.2 mL of Accutase (EMD Millipore) and neutralized with an equal volume of medium. Viable cells in each well were counted using an ORFLO Moxi Mini Automated Cell Counter (Type S cassette), and the average of the 4 wells was taken as the value for each biological replicate. Dose-response curves representing the fraction of viable cells relative to nonirradiated (NI) controls were generated for both VHEE and CIEE.

### Mouse precision-cut lung slices preparation and cell division assay

Organotypic precision-cut lung slices (PCLS) were prepared following the protocol described by Dubail et al.^25^ Lungs from anesthetized C57BL/6J mice (6−10 weeks old) were sectioned into 300μm slices using a vibratome and maintained in culture at $37^{\circ }C$ with $5\%\;CO_{2}$ until irradiation. For VHEE irradiation, 5 slices were randomly assigned to each dose groups (3, 6, and 9 Gy) and irradiated individually in Petri dishes. Irradiations were performed across 3 independent biological replicates, with 1 mouse used per day. Cell replication was assessed 24 hours postirradiation, a time point previously shown to best reflect physiological in vivo conditions,^25^ using a Click-iT chemistry protocol based on the incorporation of 5-ethynyl-2′-deoxyuridine (EdU) (BCK-EdUPro-FC647). Imaging was performed using a Nikon Spinning Disk TIRF-FRAP microscope, with approximately 5 to 7 fields of view (FOV) acquired per slice and analyzed with IMARIS (Bitplane). CIEE irradiations were conducted using the same lung slice preparation, dose grouping, and replication assessment protocol.

### Zebrafish embryo irradiation and morphologic quantification

Wild-type AB zebrafish embryos were maintained in E3 medium at $28^{\circ }C$ until irradiation. At the stage of development corresponding to 4 hours postfertilization, embryos were transferred into Eppendorf tubes (2 mL for VHEE, 0.5 mL for CIEE) filled with E3 medium and irradiated at room temperature ($22-24^{\;\circ }C$) with either VHEE or CIEE beams, at doses of 6 and 9 Gy. For each dose and irradiation modality, experiments were independently replicated on 3 days, with approximately 111 embryos irradiated with VHEE and around 70 embryos with CIEE. At 5 days postirradiation, a stage at which major organogenesis is complete^26^ and embryos are not yet considered protected under European and French animal regulations, embryos were fixed in 10% formalin and imaged using an Echo Rebel microscope (4× objective). Morphologic abnormalities were quantitatively assessed, focusing on body length and spinal curvature, which are well-established endpoints for developmental toxicity and DNA damage in zebrafish.^27^, ^28^, ^29^, ^30^ Both parameters were computed using a custom Python-based analysis tool with manual midline tracing through a graphical interface. Body length was calculated as the distance along the vertebral axis from the anterior tip of the head to the end of the vertebral column, excluding the caudal fin fold. Spinal curvature was assessed using 2 complementary metrics derived from a separately traced midline along the tail region: the mean local curvature (average angular deviation between adjacent triplets of points) and the maximum angle curvature (largest angular deviation along the spine).

### Statistical analysis

For cell viability-based survival, lung slice, and zebrafish assays, irradiated sample values were normalized to the corresponding NI control from the same experimental day and averaged across biological replicates. Dose-response curves for cell viability were fitted using the linear-quadratic model in GraphPad Prism (version 10.3.1) for visual comparison between VHEE and CIEE trends; the fit is not intended to provide quantitative α or β parameters. Differences in cell viability between the 2 beams were assessed using a 2-sided Mann-Whitney *U* test. Data distributions from lung slice and zebrafish assays are visualized using violin plots, with overlaid boxplots showing the mean, median, IQR, and whiskers extending to 1.5× IQR. Statistical comparisons for these assays were performed using the Kruskal-Wallis test to assess differences across groups, followed by Dunn's post hoc test with Holm correction for multiple comparisons (scipy.stats and scikit-posthocs, Python v3.X, SciPy v1.11.4). A 2-sided *P* value <.05 was considered statistically significant.

## Results

### Human fibroblasts exhibit comparable radiosensitivity to laser-driven VHEE and CIEE

**Figure 2:**
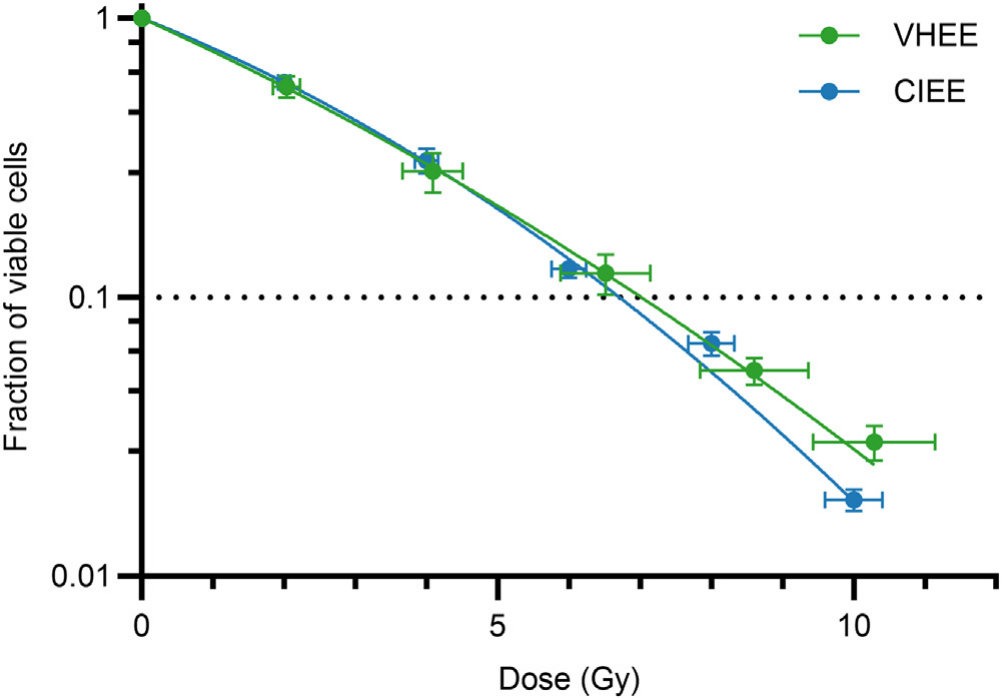
Dose-response curves for human fibroblasts (MRC5-hTERT) irradiated with either laser-driven VHEE (green) or CIEE (blue). Each point represents the mean surviving fraction of viable cells ± SEM from 3 independent biological replicates, with horizontal error bars indicating dose uncertainty. Curves were fitted using the linear-quadratic model for visualization purposes $(R^{2}>0.93$ for VHEE, $R^{2}>0.97$ for CIEE). The dotted line marks the $D_{10}$, which was not significantly different between the 2 modalities (P=.70, Mann-Whitney *U* test). Significance levels: ns, not significant; * *P* value <.05; ** *P* value <.01; *** *P* value < .001; **** *P* value < .0001. *Abbreviations*: CIEE = conventional intermediate energy electrons; VHEE = very high energy electrons.

Because no prior radiobiological experiments had been conducted using the Salle Jaune laser facility, we first assessed cell viability in MRC-hTERT human fibroblasts irradiated with the laser-driven VHEE beam. Figure 2 shows the dose-response curves for both VHEE and CIEE irradiations, fitted using the linear-quadratic model to facilitate comparison between the 2 modalities ($R^{2}=0.98$ for VHEE; $R^{2}=0.97$ for CIEE). The dose required to reduce cell viability to $10\%(D_{10})$ was (6.71±1.09) Gy for VHEE and (6.38±0.42) Gy for CIEE (uncertainty represented as standard error of the mean, SEM), with no statistically significant difference between the 2 (*P*=.70). This result indicates that VHEE and CIEE exhibit comparable radiosensitivity in this healthy cell model, further supported by the similarity in curve shapes and overlapping dose uncertainties.

### PCLS reveal similar cell division decrease after VHEE and CIEE exposure

**Figure 3:**
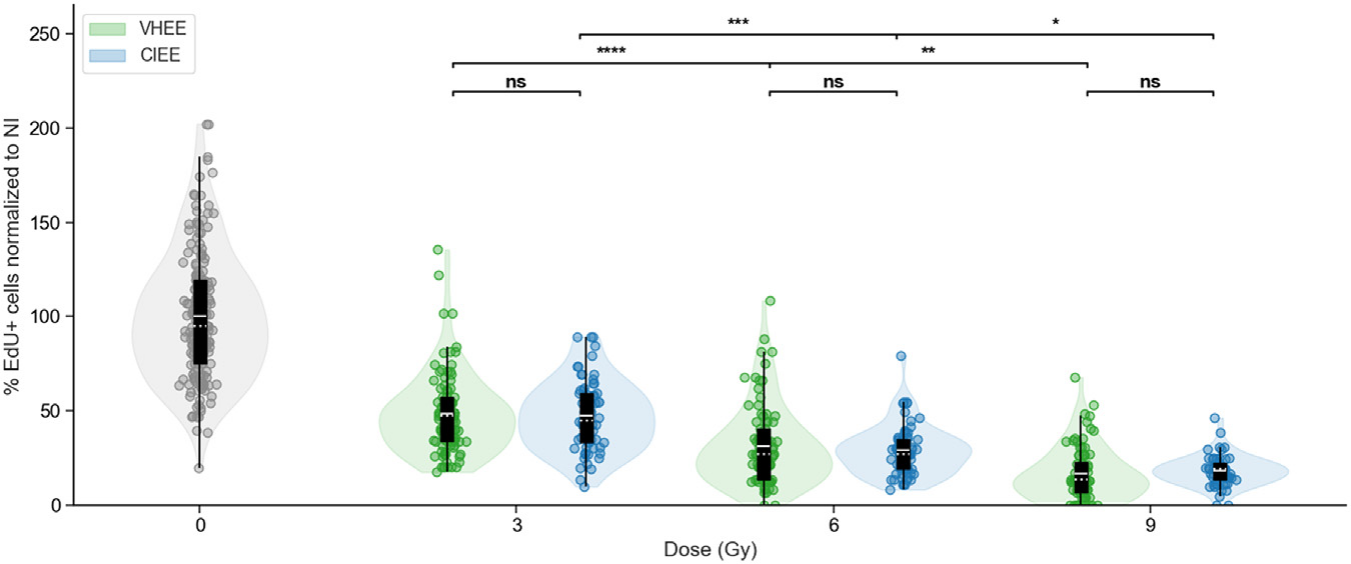
Quantification of the proportion of EdU ${\;}^{+}$cells in PCLS, normalized to NI controls, after radiation exposure to VHEE (green) or CIEE (blue) at target doses of 3, 6, and 9 Gy. Violin plots show the distribution of FOV measurements (5-7 FOV per slice) from 5 slices per dose, collected across 3 independent experimental replicates, each performed on a separate day, with overlaid boxplots (mean: solid white line; median: dotted white line; box: IQR; whiskers: 1.5× IQR). Statistical comparisons were performed using the Kruskal-Wallis test followed by Dunn's post hoc test with Holm correction. Significance levels: ns, not significant; *P value <.05; **P value <.01; ***P value <.001; ****P value <.0001. *Abbreviations*: CIEE = conventional intermediate energy electrons; EdU = 5-ethynyl-2′-deoxyuridine; FOV = fields of view; LINAC = linear accelerator; NI = nonirradiated; PCLS = precision-cut lung slices; VHEE = very high energy electrons.

To investigate beam toxicity in a tissue-relevant model, we used mouse PCLS, which preserves tissue architecture and much of the lung microenvironment. $\text{Ed}U^{+}$cell quantification following exposure to target doses of 3, 6, and 9 Gy revealed a progressive, dose-dependent reduction in cell replication for both irradiation modalities (Fig. 3). Relative to NI controls (100%), VHEE exposure reduced replication to (48.5%±2.1%),(31.6%±2.3%), and (17.0%±1.5)%(mean ±SEM) at measured doses of (3.07±0.37Gy), (5.68±0.73Gy), and (8.73±1.34Gy), respectively. CIEE yielded comparable reductions: (46.9%±2.7%), (32.2%±2.4%), and (20.0%±2.3%) (mean ± SEM) at (3.00±0.12Gy), (6.00±0.24Gy), and (9.00±0.36Gy), respectively. Interdose comparisons within each modality confirmed statistically significant reductions in replication (VHEE: $P\;\leq 1.88\times 10^{-3}$; CIEE: $P\;\leq 2.48\times 10^{-2}$). However, no significant differences were observed between VHEE and CIEE at matched dose levels, supporting their comparable biological effect in healthy lung tissue. Complete data are reported in Tables E1 and E2.

### Zebrafish embryos show comparable dose-dependent toxicity from VHEE and CIEE

**Figure 4:**
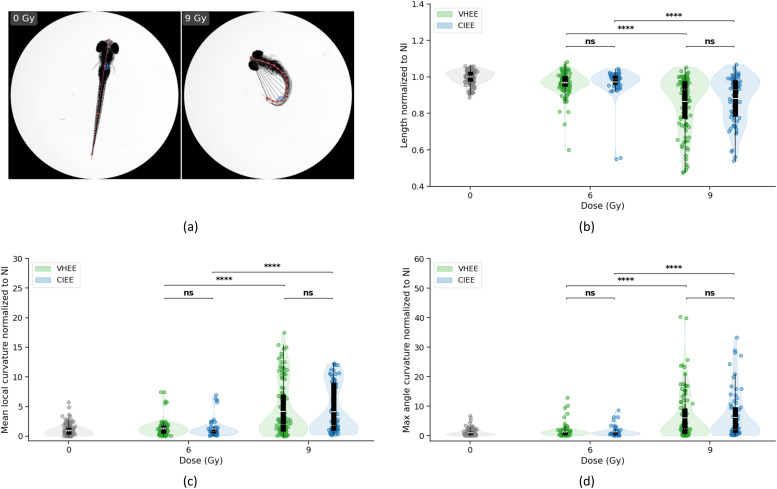
Morphologic effects of VHEE and CIEE irradiation on zebrafish embryos at 5 days postirradiation. (a) Microscope images of zebrafish at 5 days postirradiation with 0 Gy (NI) and 9 Gy VHEE, showing increased spinal curvature (curvature traces in red, maximum curvature angle in blue). (b) Normalized body length at 0, 6, and 9 Gy for VHEE (green) and CIEE (blue). (c) Normalized mean local curvature. (d) Normalized maximum angle curvature. Violin plots show full data distributions from 3 independent experimental replicates performed on different days with overlaid boxplots (mean: solid white line; median: dotted white line; box: IQR; whiskers: 1.5× IQR). Each dot represents a single embryo. Statistical comparisons: Kruskal-Wallis test with Dunn's post hoc and Holm correction. Significance levels: ns, not significant; *P value <.05; **P value <.01; ***P value <.001; ****P value <.0001 (n=111 embryos for VHEE, n=70 for CIEE). *Abbreviations*: CIEE = conventional intermediate energy electrons; LINAC = linear accelerator; NI = nonirradiated; VHEE = very high energy electrons.

Zebrafish embryos were used as an in vivo model to assess developmental toxicity following whole-body exposure to either VHEE or CIEE irradiation. Morphologic analyses at 5 days postirradiation revealed a dose-dependent effect across all evaluated metrics: normalized body length, normalized mean local curvature, and normalized maximum angle curvature (Fig. 4b-d). A progressive reduction in normalized body length was observed with increasing dose for both modalities. At 6 Gy (actual dose: (6.3 ± 0.7 Gy) VHEE, (6.00±0.24 Gy CIEE), reductions relative to NI controls were modest (−3.3% VHEE and −2.7% CIEE), whereas at 9 Gy (actual dose: (9.5±0.9Gy)VHEE, (9.00±0.36 Gy CIEE), shortening became more pronounced (−13.4% VHEE and −11.8% CIEE). Curvature metrics increased with dose: at 9 Gy, the mean local curvature reached 4.2±0.5 (VHEE) and 4.1±0.5 (CIEE), whereas the maximum angle curvature rose to 6.0±0.8 (VHEE) and 6.3±1.0 (CIEE). Statistical analysis confirmed significant intramodality effects between 6 and 9 Gy for all metrics (VHEE: $P\;\leq 2.14\times 10^{-4}$; CIEE: $P\;\leq 2.52\times 10^{-5}$). Representative images of embryos exposed to 0 Gy and 9 Gy (Fig. 4a) illustrate the curvature enhancement induced by high-dose exposure. In contrast, no statistically significant intermodality differences were observed at either 6 or 9 Gy (*P* ≥.5), supporting comparable morphologic outcomes for VHEE and CIEE. Complete data are reported in Tables E3 and E4.

## Discussion

VHEE are emerging as a promising modality for radiation therapy, offering deep tissue penetration and improved volume conformation. These characteristics make them particularly well-suited for treating tumors in anatomically complex or heterogeneous regions, where precision and robustness against tissue inhomogeneities are critical. When combined with LPA technology, VHEE delivery could become more easily integrated in hospital-scale environments, as millimeter-scale acceleration enables compact systems with reduced radioprotection requirements.

In this study, to our knowledge, we present the first multiscale radiobiological evaluation of laser-accelerated VHEE beams across in vitro, ex vivo, and in vivo models. The successful adaptation of our LPA system for biological irradiation enabled stable and reproducible beam delivery under controlled conditions, a prerequisite to translate the use of LPAs into radiobiology applications. Our results consistently show that VHEE irradiation induces biological responses similar to those observed with a conventional 7 MeV electron beam (CIEE). In human fibroblasts, dose-response curves revealed minimal differences in $D_{10}$ values between the 2 modalities, demonstrating both the feasibility of conducting radiobiological assays with our laser-driven VHEE system and the comparable toxicity of the 2 beams in this cell model. Motivated by this finding, we extended our investigation to more complex systems. Ex vivo, replication assays in organotypic mouse PCLS revealed a dose-dependent reduction in $\text{Ed}U^{+}$cells, with no statistically significant differences between the 2 beams at any dose. In vivo, zebrafish embryos, a high-throughput model sharing key anatomic, physiological, genetic, and molecular features with mammals,^31^ exhibited comparable morphologic changes following VHEE or CIEE exposure, including dose-dependent effects on body length, mean local curvature, and maximum angle curvature. Collectively, these results confirm that the comparable biological effects of VHEE and CIEE are maintained across models of increasing complexity, from single cells to whole organisms. Although our study focused on healthy tissue models and short-term endpoints, it provides a robust and controlled framework to compare the biological effects of laser-driven VHEE with those of a conventionally accelerated 7 MeV electron beam. Because these models are relatively thin, both modalities achieve full and homogeneous irradiation. One of the key advantages of VHEE, however, is their greater penetration depth and relative insensitivity to tissue heterogeneities. To fully explore this potential, future studies should involve anatomically larger systems, which would allow evaluation of spatial dose distribution in different dose delivery modalities.^1^^,^^6^^,^^7^

To advance translational relevance, future studies should incorporate more physiologically complex models, such as multicellular tumor spheroids and organoids. Spheroids reproduce essential microtumor features, including oxygen and nutrient gradients, heterogeneous proliferation zones, and intercellular communication, and their radiation responses more closely reflect those of corresponding xenografts.^32^ Organoids, derived from pluripotent or adult stem cells, recapitulate the architecture and functions of healthy or diseased human tissues, providing a powerful platform to study tissue-specific toxicity. Integrating these 3D systems with subsequent in vivo mouse studies to assess both normal tissue responses and tumor control would establish a coherent preclinical framework for the biological assessment of emerging VHEE modalities and enable direct comparison between RF- and LPA-driven sources.

Alongside increasing biological complexity, an equally important frontier lies in exploiting the distinctive beam characteristics of LPA-driven VHEE. Our system delivers subpicosecond electron bursts with instantaneous peak dose rates exceeding $10^{9}\;\text{Gy}/s$, values far beyond conventional clinical protocols. Although our current dose-per-pulse and repetition rate do not meet the parameters defining FLASH protocols, as recognized so far in the scientific literature,^33^ laser-driven ultrashort radiation pulses enable investigation of locally short, intense dose deposition and fast dose-fractionation protocols. Evidence from laser-accelerated protons has shown that temporal structuring of the dose can impair DNA repair pathways and enhance tumor cell killing, suggesting that similar mechanisms could be explored with laser-accelerated electrons. Laser-driven accelerators at higher average power and repetition rates, currently under construction worldwide, will broaden the parameter space of temporal irradiation modalities, potentially reaching FLASH-capable VHEE beams that combine a very rapid and precise temporal control with a compact accelerator design.

Within this broader context, our study positions itself in the rapidly evolving field of FLASH-VHEE and laser-driven accelerator technology for medical applications. Recent works illustrate 2 complementary research directions: Wanstall et al^11^ demonstrated FLASH sparing effects at ultrahigh dose rates in a simplified DNA model using RF-based VHEE, whereas Guo et al^22^ reported comparable tumor responses at conventional dose rates using LPA-driven VHEE. Our study starts by bridging these efforts with a systematic assessment of healthy tissue toxicity at conventional VHEE dose rates using an LPA source. This biological baseline is essential for interpreting future results in more complex systems and for designing studies that will ultimately combine advanced models with the temporal and technical capabilities unique to LPA-based accelerators. In this sense, our work establishes a foundational step linking current experimental constraints with the high therapeutic potential of next-generation, FLASH-capable laser-driven VHEE radiation therapy.

## Conclusions

To our knowledge, this study presents the first multiscale radiobiological evaluation of laser-driven VHEE across in vitro, ex vivo, and in vivo models. Through the successful adaptation of our LPA system, we achieved stable and reproducible irradiation across increasing levels of biological complexity, from fibroblast viability to tissue-level replication and whole-organism morphologic alterations. Across all systems, VHEE-induced toxicity was comparable to that of CIEE beams. These findings provide a robust foundation for future preclinical studies with VHEE beams, particularly when delivered via LPA-based systems, which offer unique advantages in compactness, flexibility, and an ultrashort temporal beam structure. Although this work focused on healthy tissues and conventional dose rates, it provides a valuable reference for future investigations into tumor-bearing models, long-term toxicity outcomes, and advanced temporal delivery strategies such as FLASH and fast fractionation. By bridging recent advances in accelerator physics with rigorous biological testing, this study positions laser-driven VHEE as a novel and promising tool in the evolving landscape of particle therapy.

## Disclosures

The authors declare that they have no known competing financial interests or personal relationships that could have appeared to influence the work reported in this paper.

## Appendix Supplementary materials

Supplements_V2.pdf Image, application 1

## Footnotes

Supplementary material associated with this article can be found in the online version at doi:10.1016/j.adro.2026.102028.
