# Supplementary material for: Multiscale Radiobiological Assessment of Laser-Driven Very High Energy Electrons Versus Conventional Electrons
Source: Adv Radiat Oncol. 2026 Mar 27;11(6):102028. doi: 10.1016/j.adro.2026.102028 (PMC13194617; doi:10.1016/j.adro.2026.102028)
Supplement: Supplements_V2.pdf [file mmc1.pdf]

SUPPLEMENTARY MATERIALS

After propagation through the vacuum–air interface, lower-energy electrons undergo diffusion under higher angles, resulting in a net spectral hardening at the target plane. To quantify the effective contribution of each energy component to the delivered dose, a Monte Carlo (Geant4) simulation was used to generate a dose-weighting curve. Multiplying this curve by the mean experimental spectrum at the source yields the effective spectrum at the biological target, with a dose-weighted mean energy of 140.6 MeV, representing the average energy of electrons contributing most to the delivered dose. The mean experimental spectrum over 60 shots (normalized), the effective spectrum at the target (normalized), and the dose-weighting curve in milli Gray at the biological sample per pico Coulomb at the source in vacuum are shown in Figure A1.

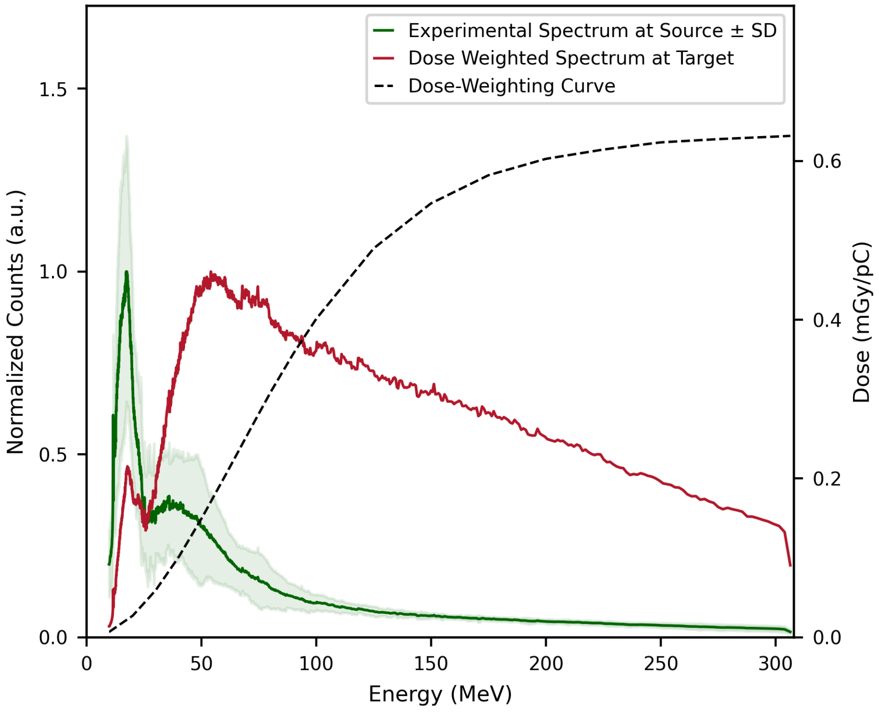

Figure A1: Normalized experimental electron spectrum at the source in vacuum (green), averaged over 60 shots, with standard deviation indicated by the shaded area. The dashed line represents the dose-weighting function from Geant4 simulations, while the red curve shows the effective spectrum at the biological target, representing the energy distribution of electrons contributing most to the delivered dose.

Table A1: Quantification of EdU^+ cells in mouse precision-cut lung slices (PCLS) following irradiation with VHEE or CIEE at different target doses. Measured doses are reported as mean ± propagated uncertainty from the three indipendent measurements. The proportion of EdU^+ cells is expressed as percentage relative to non-irradiated (NI) controls and reported as mean ± SEM.

**Modality**	**Target Dose (Gy)**	**Measured Dose (Gy) (mean ± error)**	**EdU^+ cells (% of NI) (mean ± SEM)**
CIEE	0	–	100.00 ± 3.94
	3	3.000 ± 0.12	47.62 ± 2.23
	6	6.000 ± 0.24	29.27 ± 1.57
	9	9.000 ± 0.36	18.45 ± 1.05
VHEE	0	–	100.00 ± 3.18
	3	3.072 ± 0.37	48.49 ± 2.09
	6	5.680 ± 0.73	31.55 ± 2.28
	9	8.734 ± 1.34	17.01 ± 1.53

Table A2: Statistical analysis of EdU^+ cell proportions in mouse PCLS across all irradiation conditions. A global comparison using the Kruskal–Wallis test ($H = 450.900$, $p = 2.845 \times 10^{-93}$) was followed by Dunn's post hoc test with Holm correction for pairwise comparisons. Significance levels are indicated as follows: $p < 0.05$ (*), $p < 0.01$ (**), $p < 0.001$ (***), and $p < 0.0001$ (****). Comparisons not reaching statistical significance are indicated as (ns).

**Condition A**	**Condition B**	**p-value**
0 Gy VHEE	0 Gy CIEE	1.00×10^{0} (ns)
0 Gy VHEE	3 Gy CIEE	3.72×10^{-10} (****)
0 Gy VHEE	6 Gy CIEE	4.22×10^{-27} (****)
0 Gy VHEE	9 Gy CIEE	1.00×10^{-41} (****)
0 Gy VHEE	3 Gy VHEE	2.25×10^{-12} (****)
0 Gy VHEE	6 Gy VHEE	2.00×10^{-30} (****)
0 Gy VHEE	9 Gy VHEE	1.30×10^{-53} (****)
3 Gy VHEE	3 Gy CIEE	1.00×10^{0} (ns)
3 Gy VHEE	6 Gy CIEE	1.50×10^{-4} (***)
3 Gy VHEE	9 Gy CIEE	7.46×10^{-12} (****)
3 Gy VHEE	6 Gy VHEE	8.09×10^{-5} (****)
3 Gy VHEE	9 Gy VHEE	2.04×10^{-15} (****)
6 Gy VHEE	6 Gy CIEE	1.00×10^{0} (ns)
6 Gy VHEE	9 Gy CIEE	1.21×10^{-2} (*)
6 Gy VHEE	9 Gy VHEE	1.88×10^{-3} (**)
9 Gy VHEE	9 Gy CIEE	1.00×10^{0} (ns)
3 Gy VHEE	0 Gy CIEE	2.86×10^{-9} (****)
6 Gy VHEE	0 Gy CIEE	8.61×10^{-23} (****)
9 Gy VHEE	0 Gy CIEE	5.08×10^{-40} (****)
6 Gy VHEE	3 Gy CIEE	6.26×10^{-4} (***)
9 Gy VHEE	3 Gy CIEE	1.71×10^{-12} (****)
9 Gy VHEE	6 Gy CIEE	6.19×10^{-3} (**)

Table A3: Quantification of zebrafish morphological metrics following irradiation with VHEE or CIEE at different target doses. Measured doses are reported as mean ± propagated uncertainty from the three independent measurements. Morphological metrics (normalized length, mean local curvature, and maximum angle curvature) are normalized to NI controls and expressed as mean ± SEM.

**Metric**	**Modality**	**Target Dose (Gy)**	**Measured Dose (Gy) (mean ± error)**	**Computed Metric (mean ± SEM)**
Normalized Length	CIEE	0	–	1.000 ± 0.004
	VHEE	0	–	1.000 ± 0.003
	CIEE	6	6.00 ± 0.24	0.973 ± 0.009
	VHEE	6	6.33 ± 0.68	0.967 ± 0.006
	CIEE	9	9.00 ± 0.36	0.882 ± 0.015
	VHEE	9	9.45 ± 0.90	0.866 ± 0.014
Normalized Mean Local Curvature	CIEE	0	–	1.000 ± 0.082
	VHEE	0	–	1.000 ± 0.097
	CIEE	6	6.00 ± 0.24	1.224 ± 0.165
	VHEE	6	6.33 ± 0.68	1.311 ± 0.119
	CIEE	9	9.00 ± 0.36	4.114 ± 0.494
	VHEE	9	9.45 ± 0.90	4.195 ± 0.449
Normalized Max Angle Curvature	CIEE	0	–	1.000 ± 0.103
	VHEE	0	–	1.000 ± 0.111
	CIEE	6	6.00 ± 0.24	1.359 ± 0.184
	VHEE	6	6.33 ± 0.68	1.417 ± 0.177
	CIEE	9	9.00 ± 0.36	6.270 ± 0.956
	VHEE	9	9.45 ± 0.90	6.019 ± 0.781

Table A4: Pairwise statistical comparisons of zebrafish morphological metrics across all irradiation conditions and doses. A Kruskal–Wallis test was performed for each metric: normalized length ($H = 143.72$, $p = 2.89 \times 10^{-29}$), mean local curvature ($H = 75.27$, $p = 8.16 \times 10^{-15}$), and maximum angle curvature ($H = 89.09$, $p = 1.04 \times 10^{-17}$). These were followed by Dunn's post hoc test with Holm correction for multiple comparisons. Pairwise p-values for each metric are reported in the table. Significance levels are indicated as follows: $p < 0.05$ (*), $p < 0.01$ (**), $p < 0.001$ (***), and $p < 0.0001$ (****). Comparisons not reaching statistical significance are indicated as (ns).

**Condition A**	**Condition B**	**p-value (Length)**	**p-value (Mean Curvature)**	**p-value (Max Angle Curvature)**
0 Gy VHEE	0 Gy CIEE	2.35×10^{-1} (ns)	1.00×10^{0} (ns)	1.00×10^{0} (ns)
6 Gy VHEE	0 Gy VHEE	1.87×10^{-5} (****)	9.76×10^{-2} (ns)	1.16×10^{-1} (ns)
6 Gy VHEE	0 Gy CIEE	2.02×10^{-4} (***)	1.00×10^{0} (ns)	1.00×10^{0} (ns)
6 Gy VHEE	6 Gy CIEE	4.95×10^{-1} (ns)	1.00×10^{0} (ns)	1.00×10^{0} (ns)
6 Gy VHEE	9 Gy VHEE	1.04×10^{-5} (****)	1.88×10^{-4} (***)	1.41×10^{-5} (****)
6 Gy VHEE	9 Gy CIEE	8.35×10^{-4} (***)	2.14×10^{-4} (***)	3.04×10^{-6} (****)
9 Gy VHEE	0 Gy VHEE	2.80×10^{-20} (****)	4.49×10^{-10} (****)	1.20×10^{-11} (****)
9 Gy VHEE	0 Gy CIEE	1.68×10^{-15} (****)	2.52×10^{-5} (****)	4.59×10^{-6} (****)
9 Gy VHEE	6 Gy CIEE	2.04×10^{-7} (****)	2.15×10^{-5} (****)	1.92×10^{-4} (***)
9 Gy VHEE	6 Gy VHEE	1.04×10^{-5} (****)	1.88×10^{-4} (***)	1.41×10^{-5} (****)
9 Gy VHEE	9 Gy CIEE	1.00×10^{0} (ns)	1.00×10^{0} (ns)	1.00×10^{0} (ns)
6 Gy CIEE	0 Gy CIEE	4.41×10^{-2} (*)	1.00×10^{0} (ns)	1.00×10^{0} (ns)
6 Gy CIEE	0 Gy VHEE	3.10×10^{-2} (*)	1.00×10^{0} (ns)	2.35×10^{-1} (ns)
9 Gy CIEE	0 Gy CIEE	1.30×10^{-11} (****)	2.52×10^{-5} (****)	8.92×10^{-7} (****)
9 Gy CIEE	0 Gy VHEE	2.71×10^{-14} (****)	3.43×10^{-9} (****)	7.32×10^{-12} (****)
9 Gy CIEE	6 Gy CIEE	2.58×10^{-5} (****)	2.52×10^{-5} (****)	3.31×10^{-5} (****)
